# Genomic DNA from animals shows contrasting strand bias in large and small subsequences

**DOI:** 10.1186/1471-2164-9-43

**Published:** 2008-01-25

**Authors:** Kenneth J Evans

**Affiliations:** 1 School of Crystallography, Birkbeck College, University of London, Malet Street, London, WC1E 7HX, UK

## Abstract

**Background:**

For eukaryotes, there is almost no strand bias with regard to base composition, with exceptions for origins of replication and transcription start sites and transcribed regions. This paper revisits the question for subsequences of DNA taken at random from the genome.

**Results:**

For a typical mammal, for example mouse or human, there is a small strand bias throughout the genomic DNA: there is a correlation between (*G *- *C*) and (*A *- *T*) on the same strand, (that is between the difference in the number of guanine and cytosine bases and the difference in the number of adenine and thymine bases). For small subsequences – up to 1 kb – this correlation is weak but positive; but for large windows – around 50 kb to 2 Mb – the correlation is strong and negative. This effect is largely independent of GC%. Transcribed and untranscribed regions give similar correlations both for small and large subsequences, but there is a difference in these regions for intermediate sized subsequences. An analysis of the human genome showed that position within the isochore structure did not affect these correlations. An analysis of available genomes of different species shows that this contrast between large and small windows is a general feature of mammals and birds. Further down the evolutionary tree, other organisms show a similar but smaller effect. Except for the nematode, all the animals analysed showed at least a small effect.

**Conclusion:**

The correlations on the large scale may be explained by DNA replication. Transcription may be a modifier of these effects but is not the fundamental cause. These results cast light on how DNA mutations affect the genome over evolutionary time. At least for vertebrates, there is a broad relationship between body temperature and the size of the correlation. The genome of mammals and birds has a structure marked by strand bias segments.

## Background

Because of the Watson-Crick structure of DNA – A paired with T and C with G – it is necessary that the number of As must equal the number of Ts when the bases on both strands are counted. Although, this equality does not have to be true for a single strand, Chargaff's second law refers to the equality of A/T and C/G bases on a single strand [1] and broadly speaking eukaryote genomes are free of intrastrand bias [2]. Within this broad picture, a number of exceptions have been discovered at transcription start sites in plants and fungi [3], animals [4], and splice sites [5]. Strand bias has been found for long regions of DNA around actual and putative origins of replication [6]. Analysis of nearby divergent genes has shown that both replication and transcription effects are important for strand bias in a range of eukaryotes [7]. Strand bias for transcribed regions has been ascribed to transcription coupled repair [8], but some categories of SNPs do not follow the pattern [9]. There is a weak (~0.3) correlation between expression of human genes and strand bias [10]. In human genes, the strand bias has been shown to be confined to non-coding regions and accentuated at boundary regions [11]. By reversing the argument, strand bias can be used to find transcribed regions [12]: this method predicts many more transcribed regions.

This paper returns to the question of strand bias in bulk genomic DNA, that is DNA chosen at random from the entire genome. When the base composition of stretches of a DNA strand are examined, it is found that for very long subsequences there is a strong negative correlation between (*G *- *C*) and (*A *- *T*) but for small subsequences this correlation has a small positive value. (*G *- *C*) is the number of guanine bases minus the number cytosine bases, and (*A *- *T*) is the difference between the number of adenine and thymine bases. This contrasts with animal chromosomes as a whole which have almost no strand bias. A species comparison shows that there is a strong effect for mammals and birds.

## Results and Discussion

### Results for human at various sized windows

A sample of 4000 fixed-length subsequences (also called windows) were selected at random from the human genome and the correlation of the number of (*G *- *C*) bases with the number of (*A *- *T*) bases calculated. Results for window sizes ranging from 50 bases to 2 Mb are shown in the first column of Table 1. For small windows – under 1 kb – there is a *small positive *correlation of (*G *- *C*) with (*A *- *T*) so that the strand with more Gs than Cs has on average slightly *more *As than Ts. However, for large windows an entirely different result obtains. For windows of 10 kb upwards there is a *negative *correlation between (*G *- *C*) and (*A *- *T*) and for large windows this correlation is *strongly *negative and the strand with more Gs than As is overwhelmingly more likely to have *fewer *As than Ts.

**Table 1:** Correlations in subsequences taken at random from the human genome

Window length	Correlation coefficient (*G *- *C*) v (*A *- *T*) unmasked genome	Correlation coefficient (*G *- *C*) v (*A *- *T*) masked genome
50	+0.140	+0.193
100	+0.176	+0.226
200	+0.208	+0.253
500	+0.216	+0.247
1000	+0.177	+0.232
2000	+0.109	+0.189
5000	+0.012	-0.024
10000	-0.083	-0.199
20000	-0.182	-0.398
50000	-0.342	-0.626
100000	-0.384	-0.739
200000	-0.577	-0.814
500000	-0.672	-0.875
1000000	-0.769	-0.876
2000000	-0.727	-0.868

In each case the sample size is 4000. There is a smooth transition from very small to very large windows. The results for the masked genome are more pronounced than for the unmasked genome.

**Figure 1:**
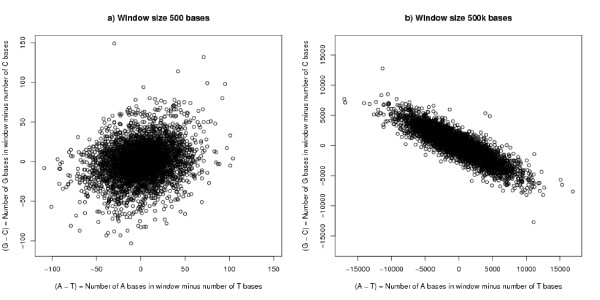
**(*G *- *C*) versus (*A *- *T*) in masked background human genome**. The Figure shows results for 4000 subsequences of length a) 500 bases and b) 500 kb taken at random from the masked human genome. The correlation coefficients are 0.247 and -0.875 respectively. The correlation for 500 kb windows taken from the chicken genome is larger than for human.

In case this result was an artefact of repeat elements in the genome, the analysis has been repeated on the sequence in which repeat elements were masked out by Ns by RepeatMasker [13]. The results are more pronounced and are shown in column two of Table 1. These results are illustrated in Figures 1a and 1b which plot (*G *- *C*) against (*A *- *T*) for windows of size 500 bases and 500 kb respectively.

**Figure 2:**
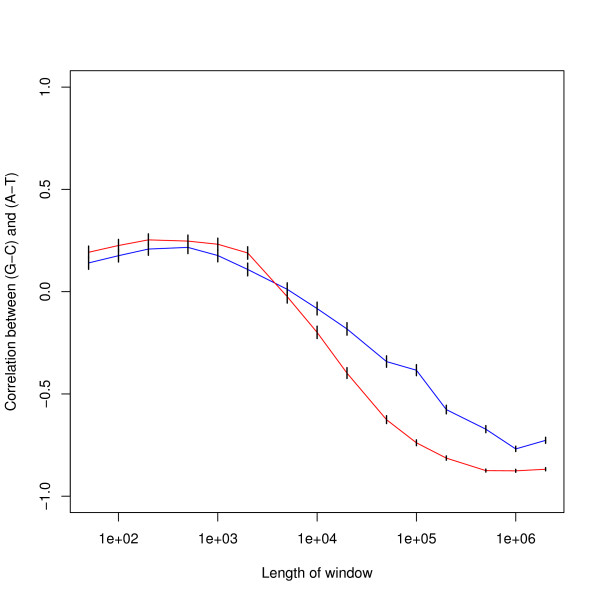
**Correlation of (*G *- *C*) versus (*A *- *T*) by window size in human genome**. The blue line shows results for the unmasked genome. The red line shows results for the masked genome and shows greater variation than for the unmasked genome. The error bars show 95% confidence intervals calculated from the Fisher transformation as plus or minus two times the standard error.

The correlation by window size given in Table 1 is also plotted in Figure 2: this Figure shows the 95% confidence limits for these correlations. Table 2 gives confidence intervals at selected values for the sample sizes used in this paper.

**Table 2:** 95% confidence limits for a quoted correlation coefficient

Quoted correlation coefficient	Lower limit n = 4000	Upper limit n = 4000	Lower limit n = 1333	Upper limit n = 1333
+0.000	-0.032	+0.032	-0.055	+0.055
+0.100	+0.069	+0.131	+0.045	+0.154
+0.250	+0.220	+0.279	+0.198	+0.301
+0.400	+0.373	+0.426	+0.353	+0.445
+0.800	+0.788	+0.811	+0.779	+0.819
+0.900	+0.894	+0.906	+0.889	+0.910
+0.950	+0.947	+0.953	+0.944	+0.955

The limits are 95% confidence intervals which have been calculated from the Fisher transformation as plus or minus two times the standard error.

The correlation coefficient is a measure of the scatter of the observations about a best fit straight line. A related but different question is the slope of this line: a note on this is given in Additional File 1.

One explanation for these results might be that in some regions of the genome there is a negative correlation and in others a positive correlation. In fact, this idea is not consistent with the analysis which is based on a random sampling of all the genome. However, to directly answer this point, a random sample of 4000 windows of size 500 kb was taken from the masked human genome and from each window a 500 base subwindow was selected, with the position chosen uniformly from the large window. Within the large and small windows the (*G *- *C*) versus (*A *- *T*) correlations are -0.87 and +0.27 respectively in line with the results in Table 1. The analysis was repeated on a subset of 1523 large windows in which the bias was comparatively large, defined by |*G *- *C*| > median {|*G *- *C*|} and |*A *- *T*| > median {|*A *- *T*|}. For this subsample of large windows the correlation is -0.95, and in the corresponding small windows the correlation is +0.19. The point of this analysis is that the correlation from the subsample of small windows is similar to that in the full sample of small windows thus proving that opposite correlations in the large and small windows can coexist within the same region of the genome.

The transition between the positive correlation and the negative correlation is very smooth and takes place over a wide range of window sizes. The essence of the explanation of how this situation can exist is that given a large window where the same strand has a surplus of Ts and Gs, there is a slight tendency for Ts and Cs to separate from the Gs and As. However, the statistics do not show a succession of small windows with clusters of these kinds in opposite orientation. The following analysis illustrates the situation with an example using a window size near the point where the correlation crosses over from negative to positive, although any window size could be used to illustrate the point. A sample of 4000 windows of length 4000 bases was taken and each window was divided into two: the upstream half and the down stream half, and various correlations were measured. Let the suffix *w *refer to the sample of windows of length 4000 bases; let the suffix 1 refer to the sample of windows obtained from the upstream half and let the suffix 2 refer to the sample of windows from the downstream half. The following correlations were found in the unmasked human genome:

a) correlation of (*G *- *C*)_*w *_with (*A *- *T*)_*w *_= 0.041

b) correlation of (*G *- *C*)_1 _with (*A *- *T*)_1 _= 0.109

c) correlation of (*G *- *C*)_2 _with (*A *- *T*)_2 _= 0.116

d) correlation of (*A *- *T*)_*w *_with (*A *- *T*)_1 _= 0.866

e) correlation of (*G *- *C*)_*w *_with (*G *- *C*)_1 _= 0.785

f) correlation of (*A *- *T*)_1 _with (*A *- *T*)_2 _= 0.506

g) correlation of (*G *- *C*)_1 _with (*G *- *C*)_2 _= 0.224

The trend shown in Table 1 implies that the correlation of (*G *- *C*) with (*A *- *T*) in the combined window (a) is different from the correlations in the subwindows (b, c), and this can be shown directly by a statistical test: correlation (a) is statistically different from correlation (b) using a Z test after a Fisher transformation n1 = n2 = 4000, Z = 3.1, two tailed test. The difference between (a) and (b, c) exists notwithstanding the high correlations at (d, e). Although all the correlations (a-g) are positive, some values (e.g. f, g) are small enough to give room for some cancellation with increasing window size, leading to the correlation of (*G *- *C*) against (*A *- *T*) decreasing as the window size increases.

### Cross species analysis

These analyses have been repeated for those species for which sequencing has progressed to the point of giving a sequence for individual chromosomes: results for unmasked and masked genomes for two sizes of windows, 500 bases and 500 kb, are given in Tables 3 and 4. The confidence intervals for these correlations can be estimated from Table 2. In each case, the sample size is 4000.

**Table 3:** Correlation of (*G *- *C*) versus (*A *- *T*) in subsequences taken at random from unmasked genomes

Scientific name	Common name	Correlation from 500 base window	Correlation from 500 kb window	Difference (Col 3 - Col 4)
*Gallus gallus*	Chicken	+0.032	-0.952	+0.983
*Homo sapiens*	Human	+0.216	-0.672	+0.889
*Pan troglodytes*	Chimpanzee	+0.233	-0.609	+0.841
*Macaca mulatta*	Rhesus macaque	+0.244	-0.696	+0.940
*Mus musculus*	Mouse	+0.158	-0.556	+0.715
*Rattus norvegicus*	Rat	+0.094	-0.608	+0.703
*Canis familiaris*	Dog	+0.301	-0.268 (A)	+0.569
*Bos taurus*	Cow	+0.306	-0.628	+0.934
*Monodelphis domestica*	Opossum	+0.501	-0.019	+0.519
*Tetraodon nigroviridis*	Puffer fish	+0.149	+0.045	+0.103
*Danio rerio*	Zebra fish	-0.045	-0.338	+0.293
*Oryzias latipes*	Medaka fish	+0.100	-0.143	+0.242
*Ciona intestinalis*	Sea squirt	-0.088	-0.277	+0.189
*Drosophila melanogaster*	Fruit fly	-0.014	-0.308	+0.295
*Anopheles gambiae*	Malaria mosquito	-0.035	-0.324	+0.289
*Caenorhabditis elegans*	Nematode	+0.313	+0.490	-0.177

In each case the sample size is 4000. The figures in the difference column have a statistically significant difference from zero, (Z test on difference between corrrelation coefficients after Fisher transformation; Z ≥ 4.7 (value for puffer fish); for example Z = 46 for human; *n*_1 _= *n*_2 _= 4000). The results are more pronounced for the higher organisms, with the result for chicken for 500 kb windows being close to 1.0. Note (A) For dog there is a small subpopulation which are outliers from the main part of the sample: when these observations are removed the correlation is -0.735. The corresponding analysis on the previous assembly gave -0.695.

**Table 4:** Correlation of (*G *- *C*) versus (*A *- *T*) in subsequences taken at random from masked genomes

Scientific name	Common name	Correlation from 500 base window	Correlation from 500 kb window	Difference (Col 3 - Col 4)
*Gallus gallus*	Chicken	-0.014	-0.964	+0.950
*Homo sapiens*	Human	+0.247	-0.875	+1.122
*Pan troglodytes*	Chimpanzee	+0.280	-0.871	+1.151
*Macaca mulatta*	Rhesus macaque	+0.306	-0.868	+1.174
*Mus musculus*	Mouse	+0.244	-0.826	+1.070
*Rattus norvegicus*	Rat	+0.282	-0.818	+1.100
*Canis familiaris*	Dog	+0.286	-0.874	+1.160
*Bos taurus*	Cow	+0.220	-0.864	+1.083
*Monodelphis domestica*	Opossum	+0.466	-0.316	+0.782
*Tetraodon nigroviridis*	Puffer fish	+0.191	+0.049	+0.142
*Danio rerio*	Zebra fish	+0.013	-0.606	+0.619
*Oryzias latipes*	Medaka fish	+0.126	-0.181	+0.307
*Ciona intestinalis*	Sea squirt	-0.116	-0.251	+0.135
*Drosophila melanogaster*	Fruit fly	+0.075	-0.056	+0.131
*Anopheles gambiae*	Malaria mosquito	+0.003	-0.452	+0.455
*Caenorhabditis elegans*	Nematode	+0.371	+0.571	-0.200

In each case the sample size is 4000. The figures in the difference column have a statistically significant difference from zero, (Z test on difference between corrrelation coefficients after Fisher transformation; Z ≥ 5.8 (value for fruit fly); for example Z = 71 for human; *n*_1 _= *n*_2 _= 4000). The difference column is more consistent than in Table 3.

The results for mammals consistently show the pattern set out above for human. The masked sequence shows a larger correlation than for wild type DNA. For the representative marsupial, the opossum, the pattern changes slightly but the difference between the correlations in the two sized windows in the masked genome of the opossum is similar to other mammals. What happens for small windows varies from species to species but between the results for small and large windows there is an additive effect which is the same for each species. The correlations for chicken are -0.95 (unmasked) and -0.96 (masked) for the 500 kb window and the fact that there is almost no correlation for the smaller windows is consistent with there being a common difference between the two windows.

The results for fish and insects are weaker and less consistent than for mammals and the details are sensitive to whether or not the correlations are based on masked DNA. Although a larger sample of species would be valuable, these results show that there is some effect at work but it is not as strong or as consistent as for mammals and birds. For both vertebrates and invertebrates, there is a positive difference between the correlation at 500 kb and 500 bases. All of the differences are statistically highly significant: the least significant difference was found for the puffer fish in unmasked DNA where the Z score is 4.7. *C. intestinalis *is representative of early chordates [14], and there is a small difference between the correlations of the two sized windows which implies that this effect arose during this era (or before) and has become larger over evolutionary time.

The clear exception to the categories described above is the more primitive *C. elegans*: in this case the correlation for 500 kb windows is positive and is greater than the correlation for the 500 base windows.

### Influence of GC%

To analyse whether the correlations are affected by the local GC%, each sample of subsequences was partitioned into three equal subsamples according to whether the GC% proportion of the subsequence was low, medium, or high. The correlation coefficients by this division by GC% for small (500 bases) and large (500 kb) window sizes for the masked genome are shown in Tables 5 and 6 respectively.

**Table 5:** Correlations by GC% subsequences taken at random from masked genomes – windows of size 500 bases

Common name	All	Low GC	Mid GC	High GC	Z value low v mid	Z value low v high	Z value mid v high
Chicken	-0.014	+0.022	-0.053	-0.010	-1.93	-0.82	+1.11
Human	+0.247	+0.194	+0.284	+0.279	+2.48	+2.32	-0.16
Chimpanzee	+0.280	+0.173	+0.343	+0.311	+4.72	+3.79	-0.93
Rhesus macaque	+0.306	+0.254	+0.352	+0.323	+2.77	+1.93	-0.84
Mouse	+0.244	+0.264	+0.213	+0.269	-1.41	+0.14	+1.55
Rat	+0.282	+0.243	+0.324	+0.292	+2.26	+1.37	-0.90
Dog	+0.286	+0.235	+0.358	+0.288	+3.50	+1.47	-2.02
Cow	+0.220	+0.206	+0.241	+0.227	+0.95	+0.58	-0.36
Opossum	+0.466	+0.444	+0.569	+0.394	+4.35	-1.56	-5.91
Puffer fish	+0.191	+0.131	+0.195	+0.241	+1.68	+2.93	+1.25
Zebra fish	+0.013	-0.087	-0.010	+0.102	+2.00	+4.90	+2.90
Medaka fish	+0.126	+0.008	+0.101	+0.218	+2.40	+5.52	+3.12
Sea squirt	-0.116	-0.169	-0.082	-0.104	+2.26	+1.71	-0.55
Fruit fly	+0.075	+0.001	+0.099	+0.107	+2.54	+2.74	+0.19
Malaria mosquito	+0.003	-0.032	-0.015	+0.051	+0.44	+2.15	+1.71
Nematode	+0.371	+0.273	+0.451	+0.376	+5.32	+2.96	-2.36

The sample size is 1333 for the subsamples determined by GC%. Confidence intervals may be estimated from Table 2. Z values are based on the difference in the correlation coefficients after Fisher transformation, n1 = n2 = 1333.

**Table 6:** Correlations by GC% subsequences taken at random from masked genomes – windows of size 500 kb

Common name	All	Low GC	Mid GC	High GC	Z value low v mid	Z value low v high	Z value mid v high
Chicken	-0.964	-0.958	-0.969	-0.966	-3.96	-2.93	+1.03
Human	-0.875	-0.844	-0.887	-0.886	-4.47	-4.35	+0.11
Chimpanzee	-0.871	-0.840	-0.884	-0.878	-4.44	-3.79	+0.65
Rhesus macaque	-0.868	-0.840	-0.872	-0.885	-3.15	-4.57	-1.42
Mouse	-0.826	-0.765	-0.862	-0.835	-7.55	-5.08	+2.46
Rat	-0.818	-0.795	-0.841	-0.815	-3.58	-1.45	+2.13
Dog	-0.874	-0.863	-0.887	-0.869	-2.65	-0.61	+2.04
Cow	-0.864	-0.874	-0.876	-0.840	-0.22	+3.31	+3.53
Opossum	-0.316	-0.138	-0.430	-0.342	-8.26	-5.60	+2.66
Puffer fish	+0.049	-0.043	+0.077	+0.107	+3.09	+3.87	+0.78
Zebra fish	-0.606	-0.685	-0.644	-0.524	+1.91	+6.62	+4.71
Medaka fish	-0.181	-0.250	-0.131	-0.165	+3.20	+2.31	-0.89
Sea squirt	-0.251	-0.460	-0.274	-0.019	+5.58	+12.34	+6.76
Fruit fly	-0.056	-0.002	+0.010	-0.156	+0.30	-3.99	-4.30
Malaria mosquito	-0.452	-0.453	-0.457	-0.453	-0.12	+0.01	+0.13
Nematode	+0.571	+0.650	+0.523	+0.562	-5.04	-3.62	+1.42

The sample size is 1333 for the subsamples determined by GC%. Confidence intervals may be estimated from Table 2. Z values are based on the difference in the correlation coefficients after Fisher transformation, n1 = n2 = 1333.

The Z values for the difference between correlations of different GC subsamples for 500 base windows are shown in Table 5. A number of values reach the traditional significance of P = 0.05 at Z = 2 (two tailed test), and for primates, e.g. human, chimpanzee, rhesus macaque, the correlation for the low GC sample is less than the mid GC sample and the low GC sample less than the high GC sample. However, the statistical results are not consistent across the species and the size of any effect is small. For the larger window size (500 kb), shown in Table 6, the statistical significance is higher and more consistent: however, for this size of window, the absolute size of the effect is very small.

The main point is that the contrast between large and small windows for the correlation coefficient does not depend on the GC%. However, there is a complex interplay between many factors that might lead to small effects: for example the effect of neighboring bases on point mutation rates (discussed below), transcription, GC% and gene placement, DNA replication: these results are therefore presented as negative controls.

### Strand bias segments

The high correlations seen in large windows for mammals and birds can only occur if the same strand has an excess of Gs and Ts for most of the sampled window. In other words, there must be long-range correlations in base composition, and correlations up to several thousand bases have already been observed [15]. The simplest situation would be where the same strand had more Ts than As for the entire chromosome, but this does not occur – the cumulative bias for an entire chromosome is small. This implies that between different regions of the genome there are break points which the long-range influence does not cross. These boundaries might either be sharp or soft: however, between these boundaries the DNA would have a given bias on the same strand: in other words, the genomes of these animals have a segmentation structure. As discussed below, a possible explanation for the long-range correlation is DNA replication which is asymmetric between the lagging and leading strands: the 5'-A+/T+-3' boundaries would correspond to origins of replication [6].

### Isochores

In the light of this conclusion, it may be asked if there is any connection with isochores. Isochores are regions of the genome where the GC% is roughly constant within the region: it has been noted that a frequency distribution of GC% in a sample of windows has a number of peaks corresponding to the GC% of the small number of types of isochores. References [16,17] discuss isochores, including the debate about the statistical reliability of isochores: these references also give maps of isochores. The isochore map of the human genome [16] has been used to analyse if strand bias depends on isochores. The boundaries of isochores in this map are given to the nearest 100 kb and therefore only results for 500 kb windows have been reported.

**Figure 3:**
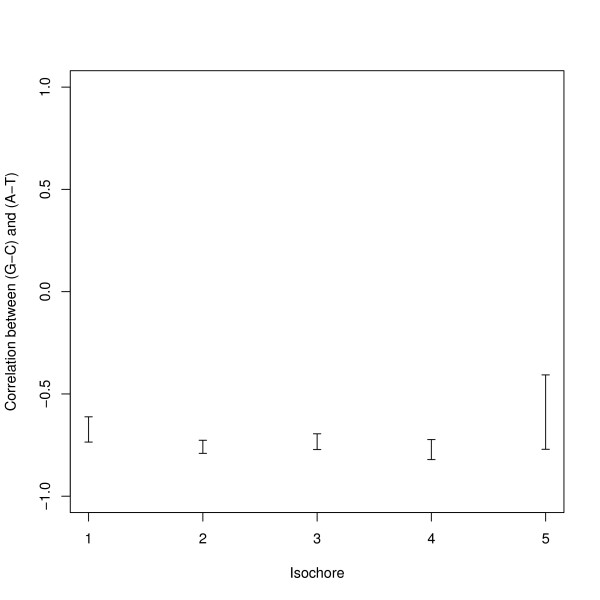
**Correlations of (*G *- *C*) versus (*A *- *T*) in 500 kb windows by isochore type in human**. This is a typical negative result of the isochore analysis. Human assembly NCBI35 = hg17 was used for this analysis and hence the average result may be slightly different from other analyses given in this paper. Isochores defined by GC% divisions at 37.5%, 41.0%, 45.5%, 51.5%. The error bars show 95% confidence intervals.

The main isochore analysis is how the correlation coefficient of (*G *- *C*) with (*A *- *T*) varies with the type of isochore: as isochore types are defined by their GC% this is also an analysis by GC% of isochore. A sample of windows of 500 kb was taken from the middle of isochores of at least this length, and allocated to one of five types of isochore using these GC% limits to distinguish the types: 37.5% 41.0% 45.5% 51.5% chosen as the midpoints of the modal values reported in [16]. The results for each subsample have been plotted in Figure 3 together with confidence limits and show no relationship with the type of isochore.

A number of other possible relationships have also been explored. Good quality isochores – that is isochores with a low variation of the GC% within the isochore-might be different from poor quality isochores. If isochores were strand bias segments then consecutive isochores should have opposite biases and so the strand bias from windows from consecutive isochores would be negatively correlated. Windows which overlapped isochore boundaries might show different results from those taken from the middle of isochores. The strand bias might depend on how close the GC% of the sample window was to the whole isochore. It is possible that the results depend on whether the isochore was a good example of its type (i.e. its GC% was close to the modal value for this type of isochore). None of these analyses suggested that there was any direct relationship between strand bias and isochores beyond that seen for the GC%. One result has been given below to summarise this group of analyses.

If isochores corresponded to strand bias segments then the strand bias on either side of the boundary would be of opposite sign. Restricting the analysis to boundaries between isochores of at least 500 kb, and taking a pair of windows each of size 500 kb either side of this boundary, the correlation between (*G *- *C*) in the two windows was +0.23. With n = 1159, the correlation coefficient is statistically significant from zero (*p *< 10^-14^), but has the wrong sign to support the hypothesis. The positive correlation between isochores suggests the opposite conclusion that isochores do not affect the strand bias. The corresponding correlation for (*A *- *T*) was also +0.23.

### Effect of transcription

**Figure 4:**
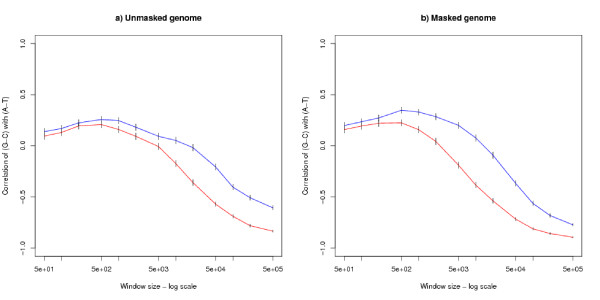
**Analysis of transcribed and non-transcribed regions in the human genome**. The graph shows correlations of (*G *- *C*) versus (*A *- *T*) by window size for a) the unmasked and b) the masked genome. For both subgraphs, each point is based on a sample of 4000 windows lying entirely within a region which is transcribed (the red lower line) or not transcribed (the blue upper line). The error bars show 95% confidence intervals.

It has been argued [4,8,10,11] that strand bias is correlated with transcription: therefore an analysis was performed on the genome after it was divided into transcribed and non-transcribed regions. For each window size, one sample was taken where each window lies entirely within a transcribed region and a second sample taken where each window lies entirely within a non-transcribed region. All samples were of size 4000. An analysis of the (*G *- *C*) versus (*A *- *T*) correlation has been made for both the unmasked and masked genomes. The results for the human genome are shown in Figures 4a and 4b. As a cross-check, the corresponding figures for mouse are given in Additional File 2.

For very large windows there are some regions which are too small for the windows to fit into. The proportion of the transcribed and untranscribed regions which can be sampled for a given window size is given in Table 7. It can be seen that this is only a consideration for the very largest windows and the window sizes plotted in Figures 4a and 4b all refer to a substantial fraction of the type of region. Similarly for large windows it is likely that different windows in the same sample will overlap. This does not affect the fairness of the estimate correlation coefficient: it does affect the estimate of the confidence intervals, but no adjustment has been made for this and it is clear that the broad conclusions are statistically sound.

**Table 7:** Proportion of human genome which can be sampled by a window of a given size

Window size	Proportion of transcibed genome	Proportion of untranscribed genome	Proportion of exon DNA	Proportion of intron DNA
50	1.000	1.000	0.994	1.000
100	1.000	1.000	0.925	1.000
200	1.000	1.000	0.691	0.997
500	1.000	1.000	0.527	0.990
1000	0.999	1.000	0.400	0.970
2000	0.998	1.000	0.232	0.923
5000	0.992	0.996	0.030	0.797
10000	0.977	0.988	0.001	0.669
20000	0.938	0.967	0.000	0.521
50000	0.809	0.900	0.000	0.312
100000	0.641	0.810	0.000	0.161
200000	0.432	0.698	0.000	0.052
500000	0.168	0.507	0.000	0.009
1000000	0.044	0.347	0.000	0.001
2000000	0.006	0.190	0.000	0.000

Based on sequence assembly NCBI35 = hg17 and ENSEMBL 33 = ENSEMBL September 2005, and coding genes.

Although Figures 4a and 4b (and the corresponding mouse figures in Additional File 2) show a difference between the transcribed and untranscribed regions, there is a noticeable similarity between the two kinds of region: the contrasting correlations observed in small and large windows is a feature of both transcribed and untranscribed regions. For small window sizes the correlations in both kinds of regions take a small positive value. For intermediate window sizes around 5000 bases upwards, there is a difference between the transcribed and untranscribed regions, with the untranscribed regions still showing a positive correlation, and the transcribed regions showing a negative correlation. For very large windows the correlations of both kinds of region are highly negative and are of similar size. At nearly all window sizes the untranscribed region shows the more positive correlation.

**Figure 5:**
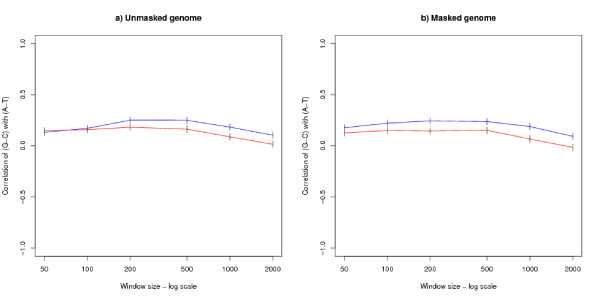
**Analysis of exons and introns in the human genome**. The graph shows correlations of (*G *- *C*) versus (*A *- *T*) by window size in a) the unmasked genome and b) the masked genome. For both subgraphs, each point is based on a sample of 4000 windows lying entirely within an intron (the blue upper line) or exon (the red lower line). The error bars show 95% confidence intervals.

Introns have also been compared with exons. Individual exons (and introns) have been kept separate and have not been fused together. Exons are comparatively short and, as Table 7 shows, it is not possible to make an intron/exon comparison for long windows. Correlations of the (*G *- *C*) versus (*A *- *T*) have therefore been calculated only for window sizes up to 2000 bases: they are shown in Figures 5a and 5b. The results for introns and exons are similar but correlations for introns are slightly more positive.

The analysis above uses the ENSEMBL definition that exons include the untranslated regions of the mRNA. A special case is the purely protein coding sequence within an individual exon: only a quarter of this type of sequence is accessible to windows 500 bases long. An analysis of windows up to this size showed no statistically significant difference from the corresponding results for exons: for example the correlation for window size 500 bases was 0.166 for the unmasked genome, and 0.176 for the masked genome (there being no statistical difference between these two figures). This analysis shows that the generally positive correlation seen for short windows is *not *the result of (a few) observations coming from coding sequences. On the contrary, in this respect, coding sequences are similar to the other types of sequence.

### Causes

The literature has pointed to two possible causes of the bias observed in this paper, transcription and replication. In the context of eukaryotes, transcription is the more discussed and replication less so. DNA replication would give the kind of strand bias found in the large scale analyses for reasons discussed previously [6]. The decisive consideration is that the strand bias occurs throughout the genome, which argues in favour of a genome wide process such as DNA replication as being the direct cause.

The strand bias observed for transcribed and untranscribed regions are similar at both ends of the graph (Figures 4a and 4b), suggesting that transcription is not the underlying cause. For intermediate sized windows, there is a marked difference between the transcribed and untranscribed regions, and this result is open to the interpretation that a transcription coupled process is an important modifier of the results at these scales. The results point towards a complicated relationship between chromatin organisation, DNA replication, transcription and strand bias [18,19].

The bias in small windows has received less attention in the literature. Again there is a small difference between transcribed and untranscribed regions but transcription (or the exon/intron split) is not the main point. It is proposed that DNA evolution has some very local process which depends on the immediate neighbouring bases, either for the insertion of individual bases or the mutation of individual bases – this would have the effect of magnifying local random strand biases. In a larger scale region consisting of one DNA replication unit (or transcription unit) there would be an additional process which would consistently give a bias in the same direction and dominate the local effect. Mutation rates of single nucleotides depend on their immediate neighbours [20,21]. Many mutations, e.g. CG to TG or CA, do not affect the correlation between (*G *- *C*) and (*A *- *T*) when both strands are considered. It is a subtle point as to which mutations do affect this correlation, and this can only be proved by a detailed simulation. However, the following argument suggests that this effect would lead to a local positive correlation. As noted above a local positive correlation can coexist with a long-range negative correlation if the Ts and Cs are (slightly) clustered together and the As and Gs (slightly) clustered together. Using the notation R = A or G, Y = T or C, the only mutations which make a difference to the number of clusters (that is runs) of Rs or Ys are those of the form YRY ↔ YYY (and the reverse complement mutations). There are sixteen of these pairs e.g. TGC ↔ TTC. Figure 4 (which refers to untranscribed regions) of [22] has been used to estimate the rates: 12 of these mutations tend to the right hand side, two to the left hand side, two are in near equilibrium. In aggregate there is therefore a slight tendency for local clustering of the Ts with the Cs.

There is a broad relationship, at least in vertebrates, between body temperature (or blood temperature) and the correlations shown in Tables 3 and 4. Body temperature is not a precisely defined variable, as it varies with position in the body, daily and yearly cycles, age of the individual, and from individual to individual. For cold blooded animals the temperature will depend on the habitat. However it is noticeable that the most negative correlation is from chicken (-0.95, -0.96) – figures in brackets are for 500 kb windows (masked, unmasked) – which has a body temperature of around 40–42°C. The next most negative group of correlations is from mammals which have a body temperature around 37°C, e.g. mouse (-0.56, -0.83). The opossum has a lower correlation (-0.02, -0.32), but its body temperature is also lower -32.3°C [23]. This trend is continued with the results of the three fish (+0.05, +0.05), (-0.34, -0.61) and (-0.14, -0.18), and also with the results from the two insects (-0.31, -0.06) and (-0.32, -0.45). It is plausible that body temperature is a causative variable as one would expect this to affect DNA mutation rates and there is a formula for mutations per site per unit time in terms of temperature and body mass [24]. The evidence suggests that the small window effect is little affected by body temperature and the large window effect is substantially affected by body temperature. The correlations from the small and large scale windows must have different causes and it is therefore plausible that the different causes are affected unequally by body temperature.

## Conclusion

There is a contrasting strand bias in base compostion for large and small subsequences of DNA sequences, with warm blooded animals showing a negative correlation for (*G *- *C*) versus (*A *- *T*) in large windows.

These correlations are independent of isochores in the human genome, and cannot be explained in terms of transcription. The strand bias can be explained by DNA replication. The results imply that the genome of mammals and birds is composed of segments of alternating strand bias. The full story is likely to be a complex interaction between chromatin architecture, DNA replication, transcription and strand bias, in line with recent interpretations [18,19] that one type of strand bias boundaries are origins of replication with both frequency of gene placement and abundance of expression related to position with respect to these boundaries.

## Methods

For the correlation analyses, and those analyses reported in Additional File 1, the sequences given in ENSEMBL [25], release 44, were used for each genome. For human this is assembly build NCBI36. Only sequences marked as chromosomes have been used – that is only those sequences have been used for which ENSEMBL gives the "id_type" as "chromosome". Windows have been taken at random from these sequences, subject to the requirement that the whole window lies within the individual sequence of this type. Where the analyses used sequences from RepeatMasker [13], the masked sequence was taken directly from ENSEMBL.

Sequences containing Ns have been used subject to the requirement that in each window the known bases must make up at least half of the window: for those analyses using paired windows the requirement applied to each window separately. This threshold was chosen because of the proportion of bases masked by RepeatMasker: for those unmasked genomes where a very stringent threshold is possible the results are not materially affected by the choice of threshold. Where a window contains unknown bases the numbers of As, Cs, Gs and Ts were grossed up so that the total A+C+G+T equalled the window size. This adjustment makes almost no difference to the results but was made to protect against variations in the number of Ns from window to window affecting the results.

The method of selecting a random window was as follows. For a given species, let *N *be the total number of bases in the genome. A random number, *r*, in the range [1, *N*] was associated with a given base in the genome, which was taken as the starting base of the window. The two strands were used alternately. Given the length of the window, the final base was then calculated. If the window did not fit on one chromosome or had too many Ns then it was rejected and a new *r *chosen.

The transcription analysis used the human assembly NCBI35, ENSEMBL release 33. Transcribed regions were identified by the information given in ENSEMBL using the start and end bases for each coding gene. This data gives the most upstream position of variant start sites and the most downstream position of variant end sites. Where genes overlap (on either strand) the transcribed regions have been fused together.

The isochore analyses used the human assembly NCBI35, ENSEMBL release 33 and the human isochore map of [16] which is based on this assembly.

For all the analyses, the computations themselves are straightforward and used C++ programs to interrogate the DNA sequences and R scripts for the statistical analyses.

## Supplementary Material

Additional file 1 **SD-ratios**. An analysis of the slope of the best fit relationship between (G-C) and (A-T). Click here for file

Additional file 2 **Figures for mouse**. Figures 4a and 4b of the main text give the results for the transcription analysis for human. Click here for file
