# Supplementary material for: Genomic DNA from animals shows contrasting strand bias in large and small subsequences
Source: BMC Genomics. 2008 Jan 25;9:43. doi: 10.1186/1471-2164-9-43 (PMC2267173; doi:10.1186/1471-2164-9-43)
Supplement: Additional file 1 — SD-ratios. An analysis of the slope of the best fit relationship between (G-C) and (A-T). [file 1471-2164-9-43-S1.pdf]

Genomic DNA from animals shows contrasting strand bias in large and small subsequences

Supplementary File 1—SD-Ratios

Kenneth J Evans

Background

The correlation coefficient, ρ, gives a measure of the uncertainty of the change in one variable with the other. A related question is how much one variable is likely to change for a unit change in the other. A standard technique for estimating this is linear regression: when y is regressed on x, the slope is given by

$$\beta = \rho \frac{S_Y}{S_X} \tag{1}$$

where S_Y and S_X are the standard deviations of the y and x values respectively. However, in the current problem there is a symmetry between the variables $(G - C)$ and $(A - T)$ and using the regression estimate would break this symmetry. The trade off between the variables has therefore been measured by using the following ratio:

$$\text{sd-ratio} = \text{sign}(\rho) \times \frac{S_Y}{S_X} \tag{2}$$

which is the geometric mean of the estimates of the $\Delta y/\Delta x$ from the two regression slopes (y on x and x on y).

Results

Results for this measure are given in Table 1 for the human genome for various window sizes and are plotted in Figure 1. In absolute value this ratio declines smoothly as the window size increases. However the notable feature is the sign reversal around 5k bases. The confidence limits for this measure can be calculated from its close relationship with the F-distribution—the upper and lower confidence limits are given by multiplying by factors which depend only on the sample size and these factors are given in Table 2.

Table 3 gives the results for different species both for large and small windows and for masked and unmasked genomes. The correlation in 500 base windows for masked genomes is very similar across species:

for chicken and the sea squirt the anomalous sign comes from the correlation coefficient which is negative but close to zero.

Discussion

Given the similarity between the correlation coefficient and the sd-ratio, it is not surprising that the sd-ratio also shows a contrast between small and large windows. The difference in the sd-ratio results from the correlation results is that the sd-ratio for small windows is almost always greater in absolute terms than for the large windows. This means that for a typical genome such as human or mouse, as the window size changes from small to large, the straight line fit changes from a relatively poor fit with a large positive slope to a relatively good fit with a smaller negative slope.

Methods

The same data sources and methods were used as for the correlation analysis of the main text.

Figure 1: SD-Ratio of $(G - C)$ and $(A - T)$ by window size in background human genome

The y axis is the ratio of the standard deviation of $(G - C)$ divided by the standard deviation $(A - T)$ in the same sample multiplied by the sign of the correlation coefficient. The blue line shows results for the unmasked genome. The red line shows results for the masked genome and shows greater variation than for the unmasked genome. The error bars show 95% confidence intervals calculated from the F distribution as plus or minus two times the standard error, on approximating the F distribution where df1 and df2 are large, df1 = df2 = (4000-1), to the normal distribution.

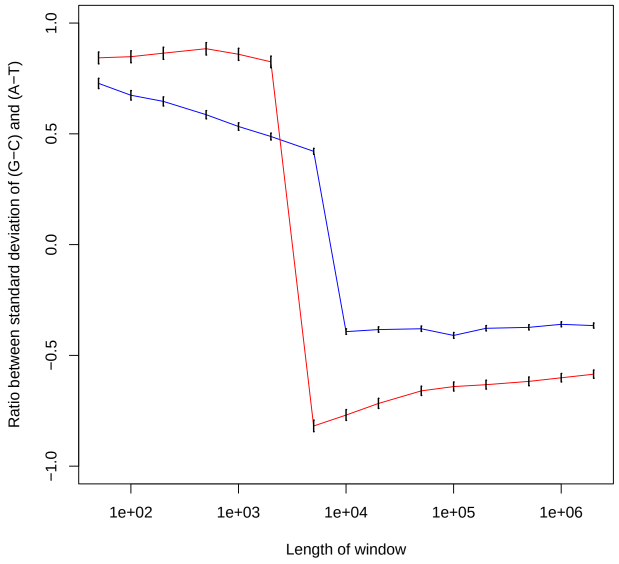

Table 1 - SD-Ratio of (G-C) and (A-T) in subsequences taken at random from the human genome

Window length	SD-ratio of $(G - C)$ and $(A - T)$ unmasked genome	SD-ratio of $(G - C)$ and $(A - T)$ masked genome
50	+0.728	+0.843
100	+0.674	+0.848
200	+0.647	+0.864
500	+0.586	+0.884
1000	+0.533	+0.859
2000	+0.488	+0.825
5000	+0.421	-0.819
10000	-0.393	-0.769
20000	-0.384	-0.717
50000	-0.380	-0.660
100000	-0.410	-0.641
200000	-0.378	-0.632
500000	-0.374	-0.617
1000000	-0.360	-0.601
2000000	-0.365	-0.585

In each case the sample size is 4000. The values shown are the ratio of the standard deviation of $(G - C)$ divided by the standard deviation $(A - T)$ in the same sample multiplied by the sign of the correlation coefficient. The absolute value of the ratio varies smoothly from very small to very large windows. However, there is a discontinuity in the sign near window sizes of 5000 bases.

Table 2 - Factors for 95% confidence limits of SD-Ratios

Sample size	Factor for lower limit	Factor for upper limit
4000	+0.968	+1.031
1333	+0.944	+1.053

Thus if in a sample of 4000 windows the sd-ratio is R then the lower confidence limit is $0.968 \times R$ and the upper confidence limit is $1.031 * R$. These confidence intervals have been calculated from the F distribution as plus or minus two times the standard error, on approximating the F distribution where df1 and df2 are large, $df1 = df2 = (n - 1)$, to the normal distribution, and noting that the F distribution refers to a ratio of variances whereas the sd-ratio is a ratio of standard deviations.

Table 3 - SD-Ratio by species

Scientific name	Common name	Unmasked 500 bases	Unmasked 500 kb	Masked 500 bases	Masked 500 kb
Gallus gallus	Chicken	+0.833	-0.706	-0.832	-0.771
Homo sapiens	Human	+0.586	-0.374	+0.884	-0.617
Pan troglodytes	Chimpanzee	+0.611	-0.391	+0.870	-0.611
Macaca mulatta	Rhesus macaque	+0.573	-0.376	+0.890	-0.611
Mus musculus	Mouse	+0.624	-0.288	+0.817	-0.530
Rattus norvegicus	Rat	+0.600	-0.265	+0.821	-0.516
Canis familiaris	Dog	+0.638	-0.473	+0.892	-0.642
Bos taurus	Cow	+0.652	-0.376	+0.838	-0.571
Monodelphis domestica	Opossum	+0.606	-0.255	+0.714	-0.370
Tetraodon nigroviridis	Puffer fish	+0.819	+0.293	+0.778	+0.275
Danio rerio	Zebra fish	-0.741	-0.469	+0.723	-0.366
Oryzias latipes	Medaka fish	+0.689	-0.334	+0.726	-0.374
Ciona intestinalis	Sea squirt	-0.699	-0.500	-0.735	-0.576
Drosophila melanogaster	Fruit fly	-0.562	-0.425	+0.614	-0.565
Anopheles gambiae	Malaria mosquito	-0.743	-0.980	+0.828	-1.168
Caenorhabditis elegans	Nematode	+0.709	+0.640	+0.707	+0.625

The results are based on a sample 4000 windows: the confidence limits may be estimated from Table 2. The outliers noted in the calculation for dog (see notes to table 3 main text) make only a small effect on the present calculation—removing them would change the figure of -0.473 to 0.421.
