# Supplementary material for: Genomic DNA from animals shows contrasting strand bias in large and small subsequences
Source: BMC Genomics. 2008 Jan 25;9:43. doi: 10.1186/1471-2164-9-43 (PMC2267173; doi:10.1186/1471-2164-9-43)

Genomic DNA from animals shows contrasting strand bias in large and small subsequences

Supplementary File 2—Figures for Mouse

Kenneth J Evans

Note

This file gives two figures for mouse corresponding to the analysis for transcribed regions for human given in the main text.

Data

The analysis used NCBIM36 for the sequence assembly and ENSEMBL release 44 for the information on gene positions.

Figure 1: Analysis of transcribed and non-transcribed regions in unmasked mouse genome

The graph shows correlations of $(G - C)$ versus $(A - T)$ by window size in the unmasked mouse genome. Each point is based on a sample of 4000 windows lying entirely within a region which is transcribed (the red lower line) or not transcribed (the blue upper line). This figure corresponds to Figure 4a of the main text.

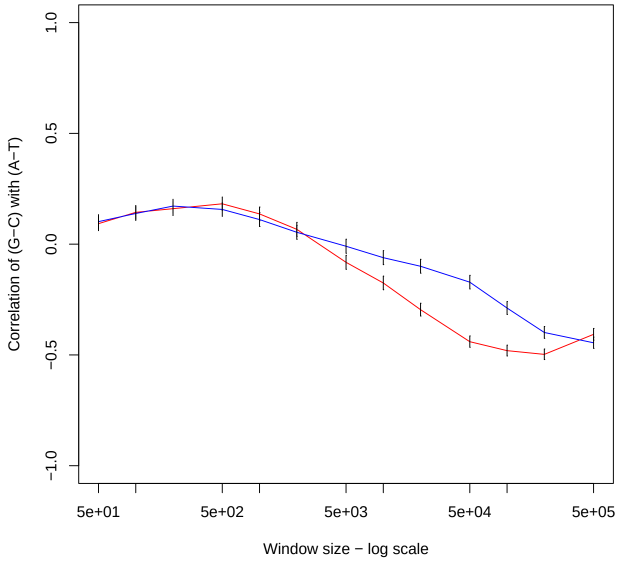

Figure 2: Analysis of transcribed and non-transcribed regions in masked mouse genome

The graph shows correlations of $(G - C)$ versus $(A - T)$ by window size in the unmasked mouse genome. Each point is based on a sample of 4000 windows lying entirely within a region which is transcribed (the red lower line) or not transcribed (the blue upper line). This figure corresponds to Figure 4b of the main text.

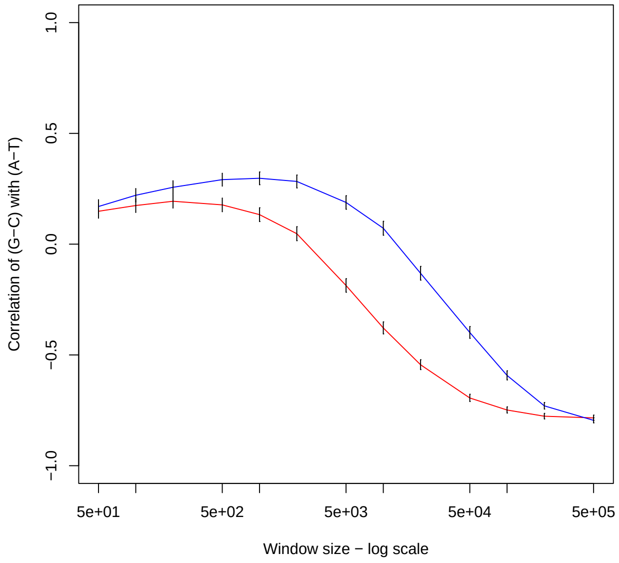

Supplement: Additional file 2 — Figures for mouse. Figures 4a and 4b of the main text give the results for the transcription analysis for human. [file 1471-2164-9-43-S2.pdf]
